# Recent advances in chromosome engineering

**DOI:** 10.1007/s10577-015-9469-5

**Published:** 2015-02-15

**Authors:** Natalay Kouprina, Vladimir Larionov

**Affiliations:** Developmental Therapeutics Branch, National Cancer Institute, Bethesda, MD 20892 USA

**Keywords:** Artificial chromosome, CRISPR, Gene therapy, Kinetochore, Stem cell, Synthetic biology, TALEN, Transgenic, ZFN

This special issue of Chromosome Research contains 12 invited topical review articles, which cover some of the most active areas of genome engineering using artificial chromosomes and engineered nucleases, zinc-finger nucleases (ZFNs), transcription activator-like effector nucleases (TALENs), and CRISPR/Cas9. This new field continues to be rapidly developed and, indeed, in the past year alone, there have been 513 papers cited in PubMed that are focused on genome/chromosome engineering in different organisms ranging from bacteria to plants and animals, as well as in cultured cells such as ES and iPS cells. These review articles were written by experts of their respective fields to bring together a vast amount of literature in a clear and didactic manner.

Four among 12 reviews are focused on the construction and applications of human and mouse artificial chromosomes (HACs or MACs). Since their description in late nineties, ACs carrying a functional kinetochore were considered a promising system for gene delivery and expression with the potential to overcome many problems inherent to viral-based gene transfer systems. Indeed, ACs avoid the limited cloning capacity, the lack of copy number control, and the insertional mutagenesis due to integration into host chromosomes that plague viral vectors. ACs can be engineered by “top-down” and “bottom-up” (or by de novo formation by centromere seeding) approaches. The top-down approach has typically involved a telomere-associated chromosome fragmentation technique in the homologous recombination-proficient chicken DT40 cell line. This approach can generate linear minichromosomes or ACs ranging in size from 0.5 to 10 Mb that retain functional centromeres and are mitotically stable in human or mouse cells. The most advanced top-down HAC in which almost all pericentromeric regions are removed was constructed in the Oshimura’s lab by several rounds of the telomere-directed breakage of human chromosome 21 (Oshimura et al. 2013). The bottom-up approach of HACs generation includes transfection of human cells with either natural higher-order repeat (HOR) or synthetic alpha-satellite (alphoid) DNA of 30–200 kb cloned into a circular BAC vector or into a linear YAC vector carrying telomeric sequences (Basu and Willard 2006). In all cases, HAC formation (a circular HAC if a BAC is used or a linear HAC if a YAC is used) was accompanied by 20–30-fold multimerization of the input DNA. Over the past 14 years, several groups have reported the successful generation of de novo constructed HACs from different substrates. The size of those HACs ranges in size from 1 to 10 Mb, and the HACs have been shown to be mitotically stable both in human and rodent cells.

Another type of de novo formed artificial chromosomes are the satellite DNA-based artificial chromosomes (SATACs) that can be made in cells of different mammalian and plant species. The in vivo generation of SATACs is based upon the induction of large-scale amplification via integration of exogenous DNA into specific chromosomal regions (Holló et al. 1996). The short arm of acrocentric chromosomes, which contains tandemly repeated ribosomal RNA genes (rDNA) and different non-coding satellite DNA sequences, is an optimal chromosomal region to induce de novo SATAC formation. Thus, almost any cultured mammalian cell line is suitable for the production of SATACs. The potential to use any mammalian cell line for SATAC formation is a major advantage of this technology over other de novo HAC and MAC technologies. Furthermore, SATACs have been purified to near homogeneity by high-speed flow sorting due to their unique nucleotide composition and later used for microinjection into the pronuclei of murine embryos to generate transgenic mice. All three described protocols generate minichromosomes with functional centromeres that maintain their nuclear location and properly segregate during mitotic and meiotic cell divisions. Several studies have demonstrated the use of top-down and de novo generated HACs and MACs for the delivery and expression of genes in human and rodent gene-deficient cell lines, the generation of iPS cells and for animal transgenesis (Kouprina et al. 2014).

In this issue, Dr. Hiroshi Masumoto and co-authors present data on molecular mechanism for de novo HAC formation from natural and synthetic alpha-satellite DNA arrays. Their data clearly demonstrate the critical role of CENP-A assembly on input DNA for de novo kinetochore formation. Using synthetic alpha-satellite DNA arrays containing tet-O sequences, this group developed the first HAC vector with a regulated centromere that was suitable for gene transfer studies and that provided a unique method to study individual human kinetochore.

In his review, Dr. Robert Katona provides a detailed description of de novo formed satellite DNA-based mammalian artificial chromosomes (SATACs) and their comparison with HACs or MACs generated by other approaches. The SATACs vectors were adapted for gene delivery and gene expression in human cells by insertion of a unique gene acceptor loxP site. The vectors were then used for both the generation of transgenic animals with stable germline transmission of SATACs and tissue-specific expression of a therapeutic gene loaded into the artificial chromosome. Similar to HACs, SATACs could be used in cell therapy protocols, where the most attractive host cells are versatile multilineage stem cells, such as adult derived early progenitor cells and embryonic stem cells that have pluripotent and self-renewing properties to address multiple disease targets.

In their review, Dr. Daniela Moralli and Dr. Zoia L. Monaco describe de novo HAC formation from input alpha-satellite DNA in human embryonic stem cells (hESc) which until recently had never been reported before. Transferring intact DNA molecules essential for HAC formation intact across the cell membrane has been a challenge for a number of years. A highly efficient delivery system based on HSV-1 amplicons was used to target DNA directly to the ES cell nucleus, and HACs were stably generated in human embryonic stem cells (hESc) at high frequency. Normally, HAC delivery to embryonic cells is performed only via carrier cells. This demonstration of efficient HAC formation in hESc may result in a new strategy for the delivery of therapeutic genes that are regulated by tissue-specific promoters to human target cells for ex vivo treatment.

It is worth noting that the delivery of transgene-loaded HACs to desired recipient cells continues to be a fundamental challenge that impedes gene function studies as well as for gene therapy. Efficiency of the HAC transfer from donor cells where the HAC was generated into the desired recipient host cells still remains problematic because of a relatively large size of the HACs. At present, the preferred method to move a HAC from donor to recipient cells is microcell-mediated chromosome transfer (MMCT), a technique developed nearly 40 years ago (Fournier and Ruddle 1977). However, the method is tedious and only a restricted number of donor cell lines, including hamster CHO and mouse A9 cells, are suitable for microcell generation. In this issue, Dr. Mitsuo Oshimura and co-authors described several modifications that increase the efficiency of HAC transfer into recipient cells. This review also summarizes the lessons learned from studies on the construction of HACs and MACs and their ability to drive exogenous gene expression in cultured cells and transgenic animals via MMCT.

Several studies have demonstrated the use of top-down and de novo generated HACs for both the delivery and expression of genes in human gene-deficient cell lines and also for animal transgenesis. A total comprehensive list of genes loaded into HAC vectors is available in the recent review by Oshimura and co-authors (Oshimura et al. 2013). Among them are a single copy, full-length genes containing all their regulatory element such as: human *GCH1*, *CFTR*, *Factor FVIII*, *beta*-*globin*, *TP53*, *HPRT*, *VHL*, *NBS1*, *CYP3A*, *IgH*, *CSN2*, *STAT3*, as well as a ~90 kb Yamanaka *OSKM* cell-reprogramming module. The best example for the correction of a gene deficiency in model mouse and patient-derived cells by a top-down generated HAC is the use of the 21HAC carrying the dystrophin (*DYS*) gene, mutations in which lead to Duchenne muscular dystrophy (DMD). This gene is extremely large, 2.4 Mb. The complete correction of a genetic deficiency was first shown in a human DMD patient-derived cell line and in iPS cells derived from DMD model (mdx) mice. Later, Dr. Francesco Tedesco and co-authors demonstrated amelioration of the dystrophic phenotype in the mdx mice using a combination of the DYS-HAC and mesoangioblasts. The genetically corrected mesoangioblasts engrafted robustly and gave rise to many dystrophin-positive muscle fibers and muscle satellite cells in dystrophic mice, leading to morphological and functional amelioration of the phenotype that lasted for up to 8 months after transplantation. The same group applied a similar strategy to human iPS cells from a DMD patient that were genetically corrected with the DYS-HAC, thus enabling mesoangioblast-like cells to differentiate from the iPSCs harboring the DYS-HAC. In this issue, Dr. Francesco Tedesco summarizes the key steps that brought human artificial chromosomes into preclinical research for Duchenne muscular dystrophy.

Two reviews examine recent innovations in chromosome engineering that promise to greatly increase the efficiency of plant breeding. With the current state of art in minichromosomes and transformation techniques in plants, it is possible to envision the production of engineered minichromosomes that contain a large multiplicity of genes conferring many different useful traits such as pest resistances, nutritional improvements, and yield enhancements. Similar to HACs, plant minichromosomes can be engineered by top-down and bottom-up approaches. Notably, the plant bottom-up approach is based on tethering of cenH3 to a predefined chromosomal site resulting in the formation of neocentromere followed by fragmentation of the dicentric chromosome. As in HACs, the constructed advanced plant minichromosomes also use site-specific recombination system (e.g., Cre/loxP) to insert a cluster of genes of interest. The sequential insertion of multiple expression modules would also be feasible by using Cre/loxP in combination with alternative site-specific recombination systems such as, for example, phiC31 integrase.

In this issue, Dr. Michael Mette and Dr. Andreas Houben described recent approaches to manipulate individual plant chromosomes in a targeted manner in order to adapt them to needs of green biotechnology. In this context, they outline the advantages of engineering minichromosomes as vectors for transgenes in contrast to conventional transformation methods and discuss the basic strategies that can be followed to customize chromosomes in plants. In another review, Dr. James A. Birchler summarizes data on epigenetic control of kinetochore in plant and describes a general approach of telomere-mediated chromosomal truncation using Ti plasmid as well as methods to recover truncation events. He specifically discusses an advantage of extra B chromosomes for minichromosome construction. B chromosomes exist in a variety of plants and animals and usually are relatively inert. Potential basic and applied applications of synthetic chromosomes are also discussed in this review.

In the past few years, new genome editing tools have been developed that cut DNA in a precise and programmable manner. These new programmable endonucleases include zinc-finger nucleases (ZFNs), transcription activator-like endonucleases (TALENs), and the most recent addition is RNA-programmed genome editors (CRISPR/Cas9). These targetable nucleases are used to induce double-strand breaks (DSBs) at specific DNA sites which are then repaired by mechanisms that can be exploited to create sequence alterations at the cleavage site. Current results indicate that these tools can be successfully employed in a broad range of organisms which empowers them to be useful tools for improving the understanding of complex physiological systems, producing transgenic animals, including creating large animal models for human diseases, creating transgenic plants, and even for treating human genetic diseases.

Also in this issue are three reviews summarizing the recent progress in genome targeting. Dr. Marc Beyer and co-authors highlight the major technological improvements for genome editing in murine oocytes which have been achieved using TALENs. They also discussed current limitations of the technology, suggested strategies to broadly apply TALENs, and described possible future directions to facilitate gene editing in murine oocytes.

Dr. Bjoern Petersen and Dr. Heiner Niemann provide an update on the use of zinc-finger nucleases (ZFNs) to modify the genome of farm animals, summarize current knowledge on the underlying mechanism, and discuss new opportunities for generating genetically modified farm animals. Transgenic farm animals, specifically the domestic pig, share many genetic, anatomical, and physiological features with humans and may thus represent a suitable model for specific diseases, including cystic fibrosis, diabetes, cancer, and several neurological disorders.

In his review, Dr. Zhongde Wang provides a comparative analysis of works on genome engineering in cattle using engineered zinc-finger nucleases (ZFNs), TALEN proteins, and CRISPR-Cas9 nucleases. Of these three, CRISPR/Cas9 seems to show the greatest promise and flexibility for genetic engineering. However, sequence requirements of the PAM sequence may constrain some applications of this technology. Moreover, additional studies are required to evaluate the specificity and toxicity of RNA-guided DNA endonucleases in vitro and in vivo. These studies on new programmable endonucleases could help to improve the design of next-generation genome editing tools.

It is well known that mosquitoes are high impact disease vectors that transmit pathogenic agents that cause diseases such as malaria, yellow fever, chikungunya, and dengue. Techniques such as the sterile insect technique and release of insects with dominant lethality are currently being investigated to control mosquito population. In this issue, Dr. Zach N. Adelman and co-authors update a progress in the use of site-specific nucleases for chromosome editing in insects. The authors emphasize that some organisms may differ from each other in efficiency of known DNA repair pathways. Therefore, more research on the peculiarities of DNA repair pathways in mosquitoes are needed to achieve more precise chromosomal engineering of this organism.

In this issue, Dr. Philip D. Weyman and co-authors from the J. Craig Venter Institute outlined recent progress in the methodology for acquiring chromosomes and chromosome-scale DNA molecules in *Bacillus subtilis*, *Escherichia coli*, and *Saccharomyces cerevisiae*. Cloning and propagating the entire genome of one bacterium within another was first demonstrated in 2005. Using yeast *S. cerevisiae* to clone whole bacterial genomes as circular yeast artificial chromosomes (YACs) was a breakthrough for synthetic biology. Yeast recombinational machinery also allows the assembling of multiple overlapping genomic fragments into entire genome in a single step by transformation-associated recombination (TAR) (Kouprina and Larionov 2006). Right now, several complete bacterial genomes (with range from 0.6 to 2.7 Mb) have been assembled by TAR into YACs that are stable propagated in yeast cells. Another advantage of yeast as a host for microbe genomes is that the cloned chromosomes or chromosome fragments can be easily manipulated using powerful yeast genetic tools. It is worth noting that yeast cells remain a unique system for selective isolation of any desirable genomic fragment or gene from total genomic DNAs, using TAR cloning protocol. So far, this is the only approach able to isolate full-length genes that are tens of kilobase in size from mammalian genomes for their complementation analysis using HAC vectors (Kim et al. 2011).

To summarize, the reviews presented here clearly demonstrate the great progress that has been accomplished in the field of genome engineering using artificial chromosomes and site-specific nucleases that give a wide spectrum of promises from development of new transgenic animal models to therapeutic use in cell therapy, gene therapy, and immunotherapy.

As guest editors, we thank all our colleagues who contributed such outstanding articles to this issue. It was such a pleasure to work with them from invitation to final production. We also acknowledge Conly Rieder and Beth Sullivan, Editor-in-Chief and Executive Editor of *Chromosome Research*, for their guidance during this endeavor and for establishing the high standards found in this journal.

## Abbreviations

ZFNs: Zinc-finger nucleases
TALENs: Transcription activator-like effector nucleases
CRISPR/Cas9: RNA-programmed genome editors
HAC: Human artificial chromosome
MAC: Mammalian artificial chromosome
SATACs: Satellite DNA-based artificial chromosomes
AH: Artificial chromosome
HOR: Higher-order repeat
YAC: Yeast artificial chromosome
BAC: Bacterial artificial chromosome
TAR: Transformation-associated recombination
hESc: Human embryonic stem cells
MMCT: Microcell-mediated chromosome transfer
DMD: Duchenne muscular dystrophy
DSBs: Double-strand breaks
CENP-A: Centromeric modified histone H3 (CENH3, Cid, Cnp1, Cse4, HCP3)
